# Residues W215, E217 and E192 control the allosteric E*-E equilibrium of thrombin

**DOI:** 10.1038/s41598-019-48839-1

**Published:** 2019-08-23

**Authors:** Leslie A. Pelc, Sarah K. Koester, Zhiwei Chen, Noah E. Gistover, Enrico Di Cera

**Affiliations:** Edward A. Doisy Department of Biochemistry and Molecular Biology, Saint Louis University School of Medicine, St. Louis, MO 63104 USA

**Keywords:** Enzyme mechanisms, X-ray crystallography

## Abstract

A pre-existing, allosteric equilibrium between closed (E*) and open (E) conformations of the active site influences the level of activity in the trypsin fold and defines ligand binding according to the mechanism of conformational selection. Using the clotting protease thrombin as a model system, we investigate the molecular determinants of the E*-E equilibrium through rapid kinetics and X-ray structural biology. The equilibrium is controlled by three residues positioned around the active site. W215 on the 215–217 segment defining the west wall of the active site controls the rate of transition from E to E* through hydrophobic interaction with F227. E192 on the opposite 190–193 segment defining the east wall of the active site controls the rate of transition from E* to E through electrostatic repulsion of E217. The side chain of E217 acts as a lever that moves the entire 215–217 segment in the E*-E equilibrium. Removal of this side chain converts binding to the active site to a simple lock-and-key mechanism and freezes the conformation in a state intermediate between E* and E. These findings reveal a simple framework to understand the molecular basis of a key allosteric property of the trypsin fold.

## Introduction

Trypsin-like proteases utilize a catalytic triad for activity, composed of the highly conserved residues H57, D102 and S195. Catalysis is assisted by several residues within the active site^1–3^. D189 at the bottom of the primary specificity pocket engages the Arg/Lys residue at the P1 position of substrate^4^. The oxyanion hole defined by the backbone N atoms of S195 and G193 stabilizes the developing partial charge on the tetrahedral intermediate during the catalytic cycle. The 215–217 segment defines the west wall of the active site and provides additional anchor points for substrate residues immediately upstream of the peptide bond to be cleaved. A peculiar property of this segment borne out from analysis of hundreds of crystal structures deposited in the Protein Data Bank (PDB) is that it can assume alternative conformations that directly influence access to the primary specificity pocket^5,6^. The D216G mutant of αI-tryptase crystallizes in the free form with the 215–217 segment in equilibrium between open and closed conformations in the same crystal^7^. The clotting protease thrombin crystallizes in the open or closed conformation depending on solution conditions^8^. A high resolution structure of chymotrypsinogen was the first to reveal two distinct conformations of the 215–217 segment in two molecules of the asymmetric unit^9^. Crystals of prethrombin-2 harvested from the same well document alternative arrangements of the 215–217 segment^10^.

The flexibility of the 215–217 segment is an intrinsic property of the trypsin fold and has functional consequences. Binding of a ligand to the active site requires the 215–217 segment to assume an “open” configuration and is precluded in the “closed” one when side chains and backbone shift to occlude access to the primary specificity pocket. Consistent with this scenario, recent rapid kinetics studies of proteases like chymotrypsin, thrombin, factor Xa and activated protein C^8,11–16^ have shown that ligand binding to the active site does not obey “induced fit”^17^, where a conformational rearrangement of the complex follows the initial binding step, but rather obeys the alternative mechanism of “conformational selection”^18^, where the ligand selects optimal conformations from a pre-existing equilibrium of closed (E*) and open (E) forms that precedes the binding step. The mechanism of conformational selection also applies to ligand binding to the zymogen^15,19^. The E* form predominates in the zymogen and is replaced by the E form in the protease. The replacement is gradual along the activation pathway, as illustrated by a recent investigation of the conversion of prothrombin to thrombin via the intermediates prethrombin-2 and meizothrombin^19^. The celebrated Huber-Bode mechanism of zymogen activation^20^ envisions a transition triggered by proteolytic cleavage in the conserved activation domain and subsequent organization of the active site region. The E*-E equilibrium layers on top of this mechanism and casts activation as a shift along a pre-existing spectrum of conformations. The new paradigm links activity to the intrinsic dynamics of the fold, which raises important questions about the structural determinants of this linkage and the mechanism underscoring the interconversion between E* and E.

A number of candidates emerge from analysis of the current structural database and three residues deserve particular attention. The highly conserved residue W215 shuttles in and out of the active site entrance and functions as a lid that closes and opens access to the primary specificity pocket^5,6^. This residue has long been considered the major structural determinant of the E*-E equilibrium, but recent studies support involvement of additional residues^21^. In the clotting protease thrombin^22^, E217 preferentially contributes to procoagulant and prothrombotic activities^23,24^, along with W215^25,26^, and may influence the E*-E equilibrium through its H-bonding interaction with the side chain of K224 that stabilizes the 215–217 segment and the E form in thrombin^27^, prethrombin-2^10^ and prothrombin^28–30^. On the other hand, E217 may stabilize the E* form by H-bonding to the active site S195 and occluding access to the primary specificity pocket, as reported recently for plasma kallikrein^31^. There is currently no crystal structure of the W215A or E217A mutants, but the double mutant W215A/E217A of thrombin crystallizes in a collapsed form similar to E*^32^ and so does the E217K mutant^33^. Residue E192 is positioned on the east wall of the active site, across from E217 and the 215–217 segment defining the west wall^27,34^. The side chain of E192 is an uncompensated negative charge that may influence the dynamics of neighbor side chains such as E217 through electrostatic coupling^35^. The conformation of the 215–217 backbone is important in catalysis^36^ and contributes to correct orientation of substrate in the active site^1–3^. It is unclear how the conformation of this backbone is linked to the orientation of the side chains of W215 and E217. Residue 216 of the 215–217 segment is a Gly.

In this study we investigate the role of the side chains of W215, E217 and E192 through Ala substitutions and rapid kinetics of binding of the irreversible inhibitor H-D-Phe-Pro-Arg-CH_2_Cl (PPACK). The structure of PPACK is similar to that of the highly specific chromogenic substrate H-D-Phe-Pro-Arg-p-nitroanilide (FPR), except for replacement of the leaving group with a CH_2_Cl that alkylates the active site residues S195 and H57. The results reveal a simple mechanism for the E*-E equilibrium and provide the starting point for additional analysis.

## Results

### PPACK binding to wild-type obeys conformational selection

**Figure 1:**
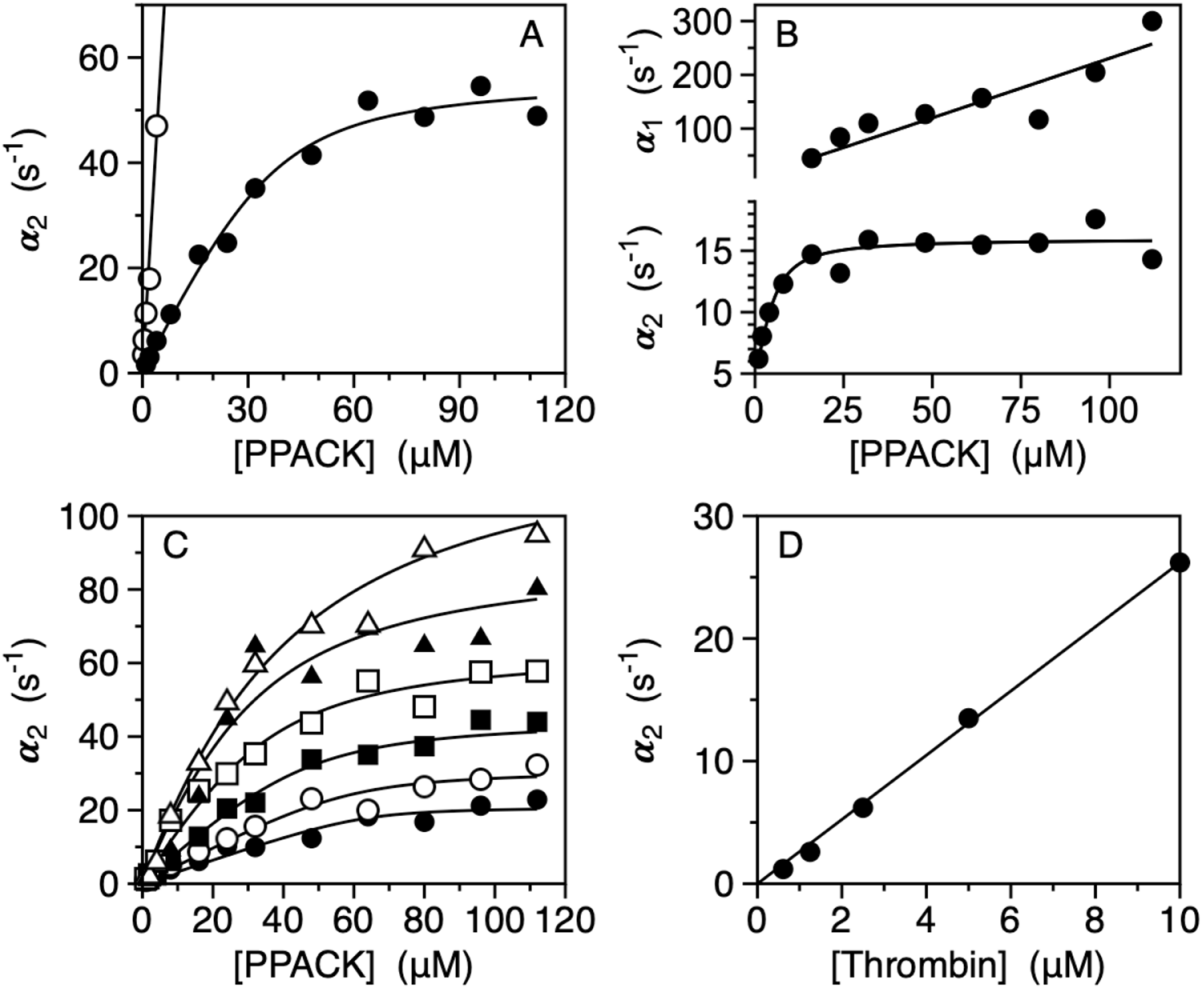
(**A**) Rapid kinetics of PPACK binding to free thrombin (closed circles) showing a single, saturable relaxation that increases hyperbolically with the ligand concentration. The continuous line was drawn according to Eq. 2 in the text with best-fit parameter values listed in Table 1. In the presence of Na^+^, the kinetic profile changes into a straight line (open circles) that obeys the lock-and-key expression^16,49^ *α* = *k*_*off*_ + *k*_*on*_[*L*], with best-fit parameter values *k*_*off*_ =  0 s^−1^ and *k*_*on*_ = 11 ± 1 μM^−1^s^−1^. (**B**) Rapid kinetics of PPACK binding to the thrombin mutant S195A showing the two relaxations predicted by the mechanism of conformational selection in Eq. 1. Continuous lines were drawn according to Eq. 2 in the text with best-fit parameter values listed in Table 1. (**C**) Rapid kinetics of PPACK binding to free thrombin over the temperature range 5–30 °C. The continuous lines were drawn according to Eq. 2 in the text with each rate constant expressed as in Eq. 3 and represent a global fit of the entire data set with best-fit parameter values: *k*_*on*_ = 1.1 ± 0.1 μM^−1^s^−1^, *k*_12_ = 45 ± 3 s^−1^, *k*_21_ =  6.4 ± 0.4 s^−1^, *E*_*on*_ = 20 ± 3 kcal/mol, *E*_12_ = 12 ± 1 kcal/mol, *E*_21_ = 37 ± 5 kcal/mol. The values of the rate constants are at the reference temperature *T*_0_ = 288.15 K (15 °C) for the sake of comparison with the data in Figs 1A and 2. Experimental conditions are: 400 mM ChCl, 50 mM Tris, 0.1% PEG8000, pH 8.0 at 5 °C (closed circles), 10 °C (open circles), 15 °C (closed squares), 20 °C (open squares), 25 °C (closed triangles), 30 °C (open triangles). (**D**) Rapid kinetics of PPACK binding to thrombin in the presence of excess enzyme. The single, saturable hyperbolic increase observed with excess ligand (**A**) becomes a simple linear relaxation in the presence of excess macromolecule if the interaction takes place according to the mechanism of conformational selection. The slope of the straight line (2.1 ± 0.1 μM^−1^s^−1^) is a measure of the *k*_*on*_ times the fraction of thrombin in the E form. The value is in good agreement with the best-fit values obtained from analysis of the data in panels A (Table 1) and C. Experimental conditions are: 400 mM ChCl, 50 mM Tris, 0.1% PEG8000, pH 8.0, at 15 °C. Data in the presence of Na^+^ (A) were obtained by replacing ChCl with NaCl in the buffer. Experimental errors for all data shown in Fig. 1A–D are 5% or less.

An informative approach to the study of ligand binding to the active site of protease or zymogen has been developed recently based on stopped flow measurements^15,19^. Extension of this approach to the case of the irreversible inhibitor PPACK allows for the study of the properties of wild-type and mutants without the need to prevent catalysis with the additional S195A replacement. Figure 1A shows the value of the slow relaxation (*α*_2_) for PPACK binding to wild-type thrombin as a function of PPACK concentration. Unlike the case of FPR binding to the S195A mutant of thrombin, where both the fast and slow relaxations in Eq. 2 could be resolved experimentally^15,19^, the fast relaxation (*α*_1_) for PPACK binding could not be detected because it is too fast to be resolved within the dead time of the stopped flow apparatus (0.5–1 ms) or spectroscopically silent. Because *α*_2_(0) = *k*_*off*_ in the mechanism of conformational selection^15,16,19,21^, a value of zero for this lower asymptote confirms the irreversible nature of PPACK inhibition. The upper asymptotic value *α*_2_(∞) = *k*_12_ measures the rate for opening of the active site in the E^*^ → *E* transition. The value 56 ± 3 s^−1^ measured for this rate constant is significantly faster than the value of 16 ± 1 s^−1^ measured for FPR binding to the S195A mutant. The difference underscores intrinsic changes in dynamics of the enzyme caused by replacement of the active site residue S195, whose importance as an end-point of allosteric transduction has been documented recently^37^. The lack of information on the fast relaxation makes it difficult to estimate the other two independent parameters *k*_*on*_ and *k*_21_ in Eq. 2. However, after constraining the value of *k*_*on*_ within a range consistent with the value measured recently for FPR binding to S195A^15,19^, a local minimum in parameter space yields a best-fit estimate for *k*_21_ = 8.8 ± 0.3 s^−1^ (Table 1). This implies that wild-type thrombin exists in equilibrium between two conformations, E* and E, that exchange over a time scale *τ* = (*k*_12_ + *k*_21_)^−1^ = 15 ms in a 1:6 ratio. FPR binding to S195A thrombin reveals an equilibrium between two conformations, E* and E, that exchange over a longer time scale *τ* = (*k*_12_ + *k*_21_)^−1^ = 56 ms in a 1:4 ratio. Replacement of S195 with Ala abrogates catalytic activity but also influences the dynamics of exchange between E* and E. Importantly, the relative distribution of these conformations is affected little and confirms a prevalence of the E conformation for the mature protease.

**Table 1:** Kinetic rate constants for PPACK binding to thrombin wild-type and mutants.

	*k*_12_ (s^−1^)	*k*_21_ (s^−1^)	*k*_*off*_ (s^−1^)	*k*_*on*_ (μM^−1^s^−1^)	*τ* (ms)	E*:E
wt	56 ± 3	8.8 ± 0.3	0 ± 0	1.5 ± 0.1	15 ± 1	1:6
S195A	16 ± 1	3.8 ± 0.8	4.6 ± 0.2	2.1 ± 0.1	51 ± 3	1:4
W215A	51 ± 3	110 ± 10	0 ± 0	1.2 ± 0.1	6.2 ± 0.3	2:1
E217A	—	—	0 ± 0	0.070 ± 0.002	—	—
E192A	11 ± 1	19 ± 1	0 ± 0	1.0 ± 0.1	33 ± 2	2:1
W215A/E217A	—	—	0 ± 0	0.00019 ± 0.00001	—	—

Experimental conditions are: 400 mM ChCl, 50 mM Tris, 0.1% PEG8000, pH 8.0, at 15 °C.

To rule out differences in the mechanism of interaction between FPR and PPACK, that share the same peptide sequence but a different C-terminal blocking group, we measured the binding of PPACK to the S195A mutant of thrombin. In this case, acylation of S195 is not possible and PPACK behaves as a reversible binder with a finite value of *k*_*off*_  = 4.6 ± 0.2 s^−1^ (Table 1) similar to that measured for the chromogenic substrate FPR. Importantly, this result holds over the short (ms) time scale of stopped flow measurements, because longer incubation (weeks) produces acylation of H57, as demonstrated by a crystal structure of the S195A mutant of meizothrombin desF1 solved in the presence of PPACK (Fig. S1 and Table S1). Binding of PPACK to the S195A mutant produces two relaxations (Fig. 1B) that can be fit to the same values of *k*_12_ = 16 ± 1 s^−1^ and *k*_21_ = 3.8 ± 0.8 s^−1^ reported for FPR binding under identical solution conditions^19^. Hence, FPR and PPACK bind with the same mechanism and with identical values of rate constants that reflect the interconversion of E* and E in the free protein.

To further validate the results obtained with PPACK binding to wild type thrombin, we measured the interaction as a function of temperature using a strategy developed for the analysis of steady state kinetics^38^. Because binding and conformational transitions in Eq. 1 have distinct activation energies, their contribution to the observed values of the relaxations in Eq. 2 changes with temperature and enables unequivocal resolution of all independent parameters involved from a global fit of the data. Data collected in the temperature range 5–30 °C (Fig. 1C) were simultaneously fit to Eq. 2 with the rate constants expressed according to the Arrhenius Eq. 3 and allowed resolution of all rate constants and their activation energies. The values obtained at the reference temperature of 15 °C are in excellent agreement with those reported in Table 1 from analysis of the data in Fig. 1A obtained independently under identical solution conditions. Resolution of the activation energies from the data in Fig. 1C provides additional details about the E*-E equilibrium, with the E*:E ratio increasing with temperature due to the higher activation energy associated with the *E* → E^*^ transition (37 ± 5 kcal/mol) compared to that of the E^*^ → *E* transition (12 ± 1 kcal/mol).

A necessary test of the validity of conformational selection requires measurements of PPACK binding under conditions where thrombin is in large excess relative to the inhibitor^15^. With excess macromolecule, the profile of *α*_2_ remains hyperbolic if binding obeys induced fit, but changes to a simple straight line in the case of conformational selection^15,39–42^. This is because a conformational transition preceding the binding step is not perturbed significantly when the macromolecule is in excess, in which case binding takes place only in the E form as a simple lock-and-key interaction^42^. Binding of PPACK under conditions where thrombin is in large excess obeys a straight line (Fig. 1D), as recently observed for FPR binding to the thrombin mutant S195A in excess concentrations^15^. Further support to the mechanism of conformational selection comes from measurements of PPACK binding in the presence of Na^+^, that is known to rigidify the fold^43,44^ and to stabilize the E form^15^. Under these conditions, PPACK binding obeys a single relaxation that increases linearly with ligand concentration (Fig. 1A), as expected for a simple lock-and-key rigid body association with a negligible rate of dissociation. The effect of Na^+^ is of physiological relevance because it counters the progressive shift of E to E* as temperature increases (Fig. 1C).

### Probing the structural determinants of the E*-E equilibrium

Having established the mechanism for PPACK binding in terms of conformational selection, we investigated the structural determinants of the E*-E equilibrium using the same strategy based upon rapid kinetics measurements. The structural database suggests that closure of the active site in the E* form is caused by the side chain of W215 coming in contact with W60d in the 60-loop and by a shift in the backbone of 215–217 that collapses the aperture leading to the primary specificity pocket^5,6^. A good measure of this aperture is the Cα-Cα distance between the highly conserved residues G193 in the oxyanion hole and G216 in the 215–217 segment that features a bimodal distribution with peaks at 8.3 Å for structures in the E* form and 12.2 Å for structures in the E form^5,6^. The aperture in the E* form is not wide enough for a ligand like PPACK to access the primary specificity pocket^5,6^. Additional steric hindrance in the E* form may come from the side chain of E192 guarding the east wall of the active site and often collapsing against the catalytic S195^33,35^, or the side chain of E217 as shown recently for plasma kallikrein^31^. When a residue occludes the active site in the E* form through its side chain, replacement with Ala is expected to change the mechanism of binding from conformational selection to a lock-and-key interaction producing a simple linear relaxation^16,45,46^. Removal of the steric hindrance in the E* form should equalize the environments of E* and E regarding accessibility of the primary specificity pocket, thereby eliminating the main functional difference between the two forms. Furthermore, the kinetic signatures of such Ala substitution should be the same as those of the E form, unless additional structural perturbations are at play such as stabilization of a new conformation intermediate between E* and E. Alternatively, the Ala replacement may result in a perturbed kinetic profile that remains hyperbolic as for the wild-type, but with a shift of the upper asymptote caused by a change in *k*_12_ and/or a change in the initial slope caused by a change in *k*_*on*_.

### Role of W215 and structure of the W215A mutant

**Figure 2:**
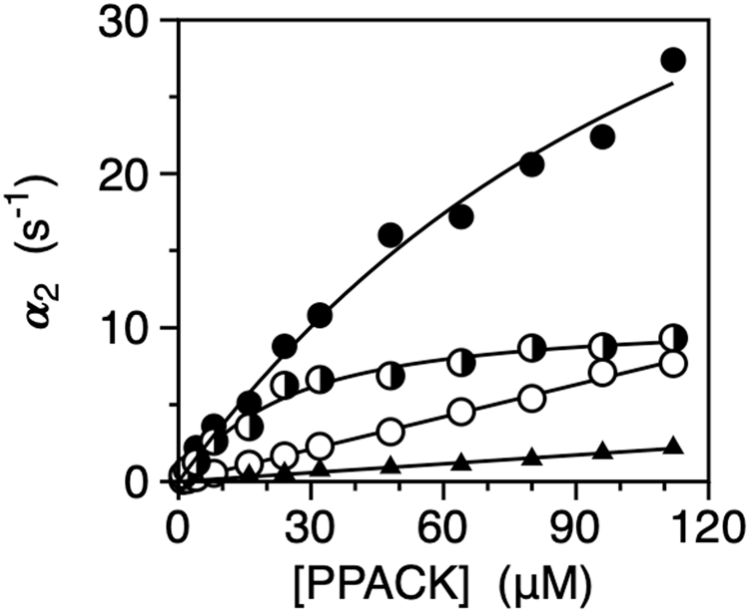
Rapid kinetics of PPACK binding to the thrombin mutants W215A (closed circles), E192A (mixed circles), E217A (open circles) and W215A/E217A (closed triangles). Binding to W215A and E192A obeys a saturable relaxation that increase hyperbolically with PPACK, as seen for wild-type (Fig. 1A). Binding to E217A and W217A/E217A obeys a simple straight line consistent with a lock-and-key mechanism of interaction. Values for W215A/E217A were increased 100-fold to enable comparison in the plot. Continuous lines were drawn according to Eq. 2 in the text (W215A and E192A) or with the lock-and-key expression^16,49^ *α* = *k*_*off*_ + *k*_*on*_[*L*] (E217A and W215A/E217A) with best-fit parameter values listed in Table 1. Experimental conditions are: 400 mM ChCl, 50 mM Tris, 0.1% PEG8000, pH 8.0, at 15 °C. Experimental errors are 5% or less.

Figure 2 shows the kinetic profile for the W215A mutant of thrombin where the role of the indole side chain is tested with an Ala replacement. Rapid kinetics of PPACK binding produce a single relaxation that increases hyperbolically with the concentration of PPACK as seen for wild-type (Fig. 1A). The profile is consistent with a pre-equilibrium between E* and E that is shifted in favor of the E* form, with a value of *k*_12_ = 51 ± 3 s^−1^ for the E^*^ → *E* transition that is comparable to that of wild-type (Table 1). The exact value of the E*:E distribution requires knowledge of *k*_21_ that cannot be resolved unequivocally from *α*_2_ only. Again, searching for a minimum around the range of values measured for wild-type gives an estimate of *k*_21_ = 110 ± 10 s^−1^ for the reverse reaction *E* → E^*^ that is > 10-fold faster than that of wild-type (Table 1). The time scale of the E*-E interconversion is *τ* = (*k*_12_ + *k*_21_)^−1^ = 6.2 ms and only slightly faster than that of wild-type. The estimated E*:E ratio for W215A is 2:1 and reversed compared to the 1:6 ratio measured for wild-type.

**Figure 3:**
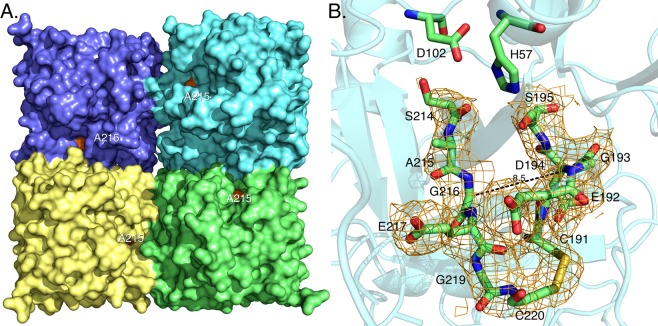
X-ray crystal structure of the thrombin mutant W215A. (**A**) Surface representation showing the architecture of the fold and the site of mutation (orange) on the west wall of the active site. (**B**) Details of the conformation of the active site region with relevant side chains and density map contoured at 1.2σ. Access to the primary specificity pocket is constrained by a collapse of the 215–217 segment that brings the Cα atoms of G193 and G216 within 8.2 Å, as typically observed in the E* form^5,6^. Details of the structure are given in Table 2.

Support to the conclusion that the W215A substitution retains the E*-E equilibrium but in a perturbed fashion that stabilizes the E* form comes from the X-ray crystal structure of the mutant (Fig. 3). Although the structure could only be solved at low resolution (Table 2), it reveals a collapsed conformation of the 215–217 segment with the Cα-Cα distance between G193 in the oxyanion hole and G216 in the adjacent 215–217 segment that shrinks to 8.5 Å. Interestingly, the perturbed environment of the active site and stabilization of a conformation similar to E* is not linked to disruption of the oxyanion hole. The backbone N atoms of G193 and S195 point in the same direction to organize a pocket for stabilization of the developing partial charge in the transition state during catalysis^1–3^. Removal of the side chain of W215 does not equalize the environment of the active site between E* and E, which would have generated a linear dependence of *α*_2_ on ligand concentration. We conclude that the role of residue W215 is to keep the active site open and to slow down the *E* → E^*^ conversion by establishing an interaction with the benzene ring of F227. Disruption of this important hydrophobic interaction accelerates closure of the active site to the E* form and results in reduced catalytic activity^25,26^. These conclusions are in agreement with recent measurements of FPR binding to the S195A/W215A mutant of thrombin^21^.

**Table 2:** Crystallographic data for the thrombin mutant W215A. ^a^Root-mean-squared deviation (Rmsd) from ideal bond lengths and angles and Rmsd in B-factors of bonded atoms. ^b^mm, main chain-main chain; ms, main chain-side chain; ss, side chain-side chain.

PDB entry	6P9U
Buffer/salt	10 mM ZnSO_4_, 100 mM MES, pH 6.5
PEG	550 MME (25%)
Data collection:	
**Wavelength (Å)**	1.5418
Space group	P2
Unit cell dimensions (Å)	a = 136.3, b = 44.2, c = 136.2, β = 90.04
Molecules/asymmetric unit	4
Resolution range (Å)	40–3.3
Observations	92698
Unique observations	22632
Completeness (%)	90.4 (90.6)
R_sym_ (%)	17.1 (69.5)
I/σ(I)	5.8 (2.0)
**Refinement:**	
Resolution (Å)	40–3.3
R_cryst_, R_free_	0.23, 0.31
Reflections (working/test)	21544/1087
Protein atoms	8942
Solvent molecules	7
Zn^++^ ions	7
Rmsd bond lengths^a^ (Å)	0.015
Rmsd angles^a^ (°)	1.8
Rmsd ΔB (Å^2^) (mm/ms/ss)^b^	4.39/3.57/4.13
<B> protein (Å^2^)	68.5
<B> Zn^++^ ions (Å^2^)	67.2
<B> Solvent (Å^2^)	54.5
**Ramachandran plot:**	
Most favored(%)	98.2
Generously allowed (%)	1.2
Disallowed (%)	0.6

### Role of E192

Residue E192 is an uncompensated negative charge guarding access to the primary specificity pocket on the segment defining the east wall of the active site^27,34^. Replacement of E192 has been reported to change the substrate specificity of thrombin^47,48^ and may perturb the E*-E transition by altering the electrostatic balance around the active site^35^. Rapid kinetics of PPACK binding to the E192A mutant produce a single relaxation that increases hyperbolically with the concentration of PPACK (Fig. 2) as seen for wild-type (Fig. 1A). The value of *k*_12_ = 11 ± 1 s^−1^ for the E^*^ → *E* transition is significantly slower than that of wild-type, but the value of *k*_21_ = 19 ± 1 s^−1^ for the reverse reaction *E* → E^*^ is 2-fold faster. The time scale of the E*-E interconversion is *τ* = (*k*_12_ + *k*_21_)^−1^ = 33 ms and similar to that of wild-type. The resulting E*:E ratio reverses from 1:6 in the wild-type to 2:1 in the mutant, again due to stabilization of the E* form. Removal of the side chain of E192 does not equalize the environment of the active site between E* and E, as seen for the W215A substitution, but causes the active site to open slower and to close slightly faster than in the wild-type. The effect is consistent with an electrostatic clash with a neighboring negatively charged side chain.

### Role of E217

A likely candidate for this electrostatic clash is E217, a highly flexible residue that moves inside the active site in kallikrein^31^. Interestingly, rapid kinetics of PPACK binding to the E217A mutant produce a profile that obeys a single linear relaxation (Fig. 2) as expected for a rigid body lock-and-key mechanism of binding that does not involve conformational transitions^16,49^. The profile is drastically different from that of wild-type and indicates that removal of the side chain of E217 freezes the conformation into a state intermediate between E* and E to which PPACK binds with a *k*_*on*_ = 0.070 ± 0.002 μM^−1^s^−1^ (Table 1). The value is > 20-fold slower than that of wild-type and not consistent with the interaction with a conformation like E. We conclude that the side chain of E217 is a major structural determinant of the E*-E equilibrium and functions as a lever that triggers opening and closing of the access to the primary specificity pocket through an electrostatic crosstalk with E192. The more pronounced perturbation of the kinetic profile observed with the E217A substitution compared to the E192A mutation is explained by E217 being part of the critical 215–217 segment that moves during the E*-E transition. Without the driving force provided by E217 in its electrostatic clash with E192, the 215–217 segment freezes into an intermediate state that compromises but does not abolish access to the primary specificity pocket.

A similar scenario is observed for the double mutant W215A/E217A where two critical residues of the 215–217 segment are replaced by Ala^50^. The mutant was originally engineered to enhance the anticoagulant properties of the individual substitutions of W215^25^ and E217^23,24^. W215A/E217A has progressed through pre-clinical^51–58^ and Phase 1 (NCT03453060) studies and will soon start Phase 2 testing (NCT039638950). Rapid kinetics of PPACK binding to the W217A/E217A double mutant produce a profile similar to that observed for the E217A mutant (Fig. 2). The combined removal of the side chains of W215 and E217 freezes the E*-E equilibrium into an intermediate state to which PPACK binds with a drastically reduced value of *k*_*on*_ = 190 ± 10 M^−1^s^−1^, which is >350-fold slower than that of E217A and almost 8,000-fold slower than that of wild-type (Table 1). Several structures of the W215A/E217A support this conclusion and reveal a collapsed conformation of the 215–217 segment^32,59^ that greatly compromises binding to the primary specificity pocket.

## Discussion

A number of high resolution crystal structures^5,6^ and rapid kinetics studies^15,19^ support the conclusion that the trypsin fold recognizes ligand at the active site according to the mechanism of conformational selection. A pre-existing equilibrium between E and E* forms controls activity in the protease and defines a key property of the fold as it transitions from zymogen to mature enzyme. The work recently carried out for thrombin and its precursors prothrombin, prethrombin-2 and meizothrombin illustrates this scenario and offers a template for the analysis of other biological systems^19^. The zymogen exists predominantly in the E* form and transitions gradually to the E form as the fold matures^19^. A similar transition from E* to E likely takes place for poorly active proteases upon interaction with specific cofactors, as observed in clotting factor VIIa^60,61^ or complement factor D^62^. Proteases can be engineered into allosteric switches that transition from E* to E on demand^63,64^, as demonstrated by mutants of clotting factor Xa that bypass the intrinsic pathway of coagulation and ameliorate hemophilia conditions^65^, or mutants of thrombin^23–26^ that are effective in the treatment of thrombotic complications and stroke. Finally, a mutation that stabilizes E* may compromise activity without direct interference with active site residues or catalysis, thereby offering context to better understand the molecular origin of pathologic phenotypes^66^. The relative distribution of E* and E offers a molecular framework to interpret many aspects of protease function^66,67^, as well as key properties of the zymogen. The ability to autoactivate observed in proprotein convertases^68–70^, plasma hyaluronan-binding protein^71^, factor VII^72^, matriptases^73,74^, prethrombin-2 and protein C^75,76^ should be cast in the context of the E*-E equilibrium, and so should the activating effect of streptokinase on plasminogen^77^, staphylocoagulase on prothrombin^78^, and of ad hoc peptides on hepatocyte growth factor^79,80^. In all of these systems, a relevant question to be asked is what are the structural determinants of the E*-E equilibrium and how the equilibrium can be perturbed to inhibit or activate function for translational applications. The results presented here provide valuable new insights.

We hypothesize that three critical residues decorating access to the active site control the E*-E equilibrium of thrombin. Their roles differ and target specific aspects of the equilibrium. The side chain of W215 keeps the active site open by interacting with F227 and slows down the transition from E to the E* form. The side chain of E192 keeps the active site open by accelerating the transition to the E form through electrostatic repulsion of the neighbor side chain of E217. Residues W215 and E192 altogether stabilize the E form by influencing the two rates governing the E*-E equilibrium, with W215 influencing *k*_21_ through hydrophobic coupling with F227 and E192 influencing *k*_12_ by electrostatic repulsion of E217 anchored to the 215–217 segment. The side chain of E217 is under the influence of both W215 and E192 and functions as a lever promoting the E*-E equilibrium. Removal of this side chain freezes the equilibrium into an intermediate state and locks the 215–217 segment into a partially collapsed conformation that greatly reduces but does not abrogate access to the primary specificity pocket. Additional components may influence the E*-E equilibrium and future mutagenesis studies will provide details. However, residues W215, E217 and E192 likely represent the end points of a molecular mechanism that opens and closes access to the primary specificity pocket. These are the three key players that control the E*-E equilibrium in thrombin according to the model proposed here.

Whether the mechanism proposed for thrombin applies to other members of the trypsin family of proteases and zymogens remains to be established by future studies. Residue 215 is highly conserved as Trp in the trypsin family (93% of the cases), but residues 217 and 192 show great variability. Specifically, residue 217 is most commonly Glu (41%), followed by Ser (29%) and Tyr (8%). Residue 192 is most commonly Gln (52%), followed by Glu (28%) and Lys (9%). Trypsin and chymotrypsin both carry W215, but residues 217 and 192 are Tyr and Gln in trypsin or Ser and Met in chymotrypsin. Preliminary analysis of PPACK binding to rat trypsin shows a phase that is too fast to resolve by stopped flow, underscoring a mechanism of recognition that differs from that of thrombin. The presence of E217 and E192 supports a mechanism for the E*-E transition as reported in the present study, but in other cases the E*-E equilibrium may be controlled by different mechanisms or may be frozen in an intermediate conformation as seen for the E217A mutant of thrombin.

A final comment should be made regarding the effect of Na^+^ on the E*-E equilibrium (Fig. 1A). Temperature studies (Fig. 1C) resolve the kinetic rate constants and activation energies associated with the scheme in Eq. 1 and predict an E*:E ratio that changes from 1:6 at 15 °C to 3:1 at 37 °C. Hence, 75% of free thrombin exists in the closed E* form at physiological temperature and would not be able to effectively cleave the procoagulant substrate fibrinogen and the prothrombotic substrate PAR1. Nearly full activity under physiological conditions is ensured by the binding of Na^+^ and conversion of E* to the active E form (Fig. 1A), an important effect that directly opposes the effect of temperature on the E*-E equilibrium. Future studies on cognate trypsin-like proteases that bind Na^+^ ^81,82^ will reveal if their mechanism of ligand recognition obeys conformational selection with kinetic features similar to those reported here for thrombin and whether Na^+^ plays a similar physiologically important role.

## Methods

### Reagents

Thrombin wild-type and its mutants W215A, E217 and E192 were expressed as prethrombin-2, purified and activated as previously described^10,30^. The irreversible inhibitor H-D-Phe-Pro-Arg-CH_2_Cl (PPACK) was purchased from Haematological Technologies. PPACK is a relevant probe of the active site as documented by detailed structural information^27,34^.

### Stopped-flow experiments

Rapid kinetic experiments of PPACK binding were conducted on an Applied Photophysics SX20 stopped-flow spectrometer using an excitation of 295 nm and a cutoff filter at 320 nm. The dead time of the mixing cell for this instrument is 0.5–1 ms. Final concentrations of 150–250 nM thrombin wild-type or mutants were used in buffer containing 50 mM Tris, 0.1% PEG8000, 400 mM ChCl, pH 8.0, at 15 °C. The solution containing the protein was mixed 1:1 with 60 µL solutions of PPACK in the same buffer. Baselines were measured by mixing the protein into buffer in the absence of ligand. Each kinetic trace was taken as the average of at least ten determinations and fit to single or double exponentials based on the analysis of residuals using software supplied by Applied Photophysics. Values of the relaxations for single and double exponential fits were derived from at least three independent titrations.

### X-ray studies

Crystallization for the human thrombin mutant W215A was achieved at 20° C by the vapor diffusion technique, using the Art Robbins Instruments Phoenix^TM^ liquid handing robot with 20 mg/ml protein 0.3ul mixed with an equal volume reservoir solution. Optimization of crystal growth was achieved by the hanging drop vapor diffusion method mixing 3 ul of protein with equal volumes of reservoir solution (Table 1). Crystals were grown in 1 week at 20° C and frozen in the solution of 10 mM ZnSO_4_, 100 mM MES, pH 6.5 and 40% PEG 550 MME. X-ray diffraction data were collected at 100° K with a home source (Rigaku 1.2 kw MMX007 generator with VHF optics) Rigaku Raxis IV^++^ detector and were indexed, integrated and scaled with the HKL2000 software package^83^. Structure was solved by molecular replacement using PHASER from the CCP4 suite^84^ and the structure of slow form of thrombin bound with PPACK (PDB entry 1SHH) as starting model. Refinement and electron density generation were performed with REFMAC5 from the CCP4 suite. 5% of the reflections were randomly selected as a test set for cross-validation. Model building and analysis were carried out using COOT^85^. Twinned crystals were detected and the refinement was performed by twin lows. Ramachandran plot was calculated using PROCHECK^86^. Statistics for data collection and refinement are summarized in Table 2. Atomic coordinates and structure factors have been deposited in the PDB (accession code: 6P9U). A structure of the meizothrombin desF1 mutant S195A bound to PPACK was solved at 2.4 Å resolution (Fig. S1, Table S1) and deposited in the PDB (accession code: 6PX5). The structure offers direct evidence that PPACK acylates H57 even in the presence of the S195A mutation (Fig. S1). However, the irreversible reaction is too slow to resolve within the short time (1–3 ms) of stopped flow measurements that detect binding of PPACK as being reversible and with a finite value of *k*_*off*_ (Fig. 1B).

### Mechanism of binding

A detailed discussion of ligand binding mechanisms studied by rapid kinetics is given elsewhere^16,21,45^, and is summarized below for the special case of irreversible binding. The relevant kinetic scheme for ligand binding to the active site of a trypsin-like protease or zymogen is the conformational selection mechanism

1 $${E}^{\ast }\begin{array}{c}{k}_{12}\\ \rightleftarrows \\ {k}_{21}\end{array}E\begin{array}{c}{k}_{on}[{\rm{L}}]\\ \rightleftarrows \\ {k}_{off}\end{array}E:\,L$$

E* and E depict the pre-existing conformations with active site accessible (E) or inaccessible (E*) to ligand binding that interconvert with first-order rate constants *k*_12_ and *k*_21_. The ratio *k*_21_/*k*_12_ gives the E*:E partitioning between the two forms (Table 1). The ligand, L, selectively binds to E with a second-order rate of association *k*_*on*_ and dissociates with a first-order rate *k*_*off*_. Under conditions where L is in large excess over the macromolecule, the reaction scheme in Eq. 1 gives two independent rates of relaxation to equilibrium according to the expression

2 $$2{\alpha }_{1,2}={k}_{12}+{k}_{21}+{k}_{on}[{\rm{L}}]+{k}_{off}\pm \sqrt{{({k}_{on}[{\rm{L}}]+{k}_{off}-{k}_{12}-{k}_{21})}^{2}+4{k}_{21}{k}_{on}[{\rm{L}}]}$$

The fast relaxation, *α*_1_ (+sign in Eq. 2), reflects the binding event and eventually grows linearly with [L]. The slow relaxation, *α*_2_ (− sign in Eq. 2) always saturates for high [L] and reflects the conformational transition associated with binding. Depending on the sign of the expression *k*_*off*_ − *k*_12_, the value of this relaxation hyperbolically decreases (*k*_*off*_ > *k*_12_) or increases (*k*_*off*_ < *k*_12_) with [L], and remains constant when *k*_*off*_ = *k*_12_^16,21,45^. Such property is exclusive of conformational selection and makes this mechanism far more general than induced fit, for which the slow relaxation can only increase hyperbolically with [L]. Indeed, the whole kinetic repertoire of induced fit is recapitulated by conformational selection as a mathematical special case^21^, which makes it necessary to distinguish between the two mechanisms every time experimental measurements of *α*_2_ produce a hyperbolic increase with [L]. The need becomes especially obvious when dealing with irreversible inhibitors such as PPACK where *k*_*off*_  = 0. PPACK possesses structural determinants for high affinity binding^27,34^. Its P1 Arg residue makes a strong ionic interaction with D189 at the bottom of the primary specificity pocket, Pro at the P2 position fits snugly against the hydrophobic surface of the 60-loop and Phe in the D enantiomer at the P3 position makes a strong edge-to-face interaction with W215 defining the western wall of the active site. Replacement of any residue of PPACK or thrombin residues interacting with PPACK significantly compromises binding^26,87–89^.

When only the slow relaxation *α*_2_ is accessible experimentally, it becomes difficult to resolve all four independent parameters in Eq. 2. The complication is circumvented by measurements carried out as a function of termperature, as originally shown for the analysis of steady state kinetics^38^. Each rate constant in Eq. 2 can be expressed in terms of its temperature dependence according to the Arrhenius equation

3 $$k={k}_{0}\exp \{-\frac{E}{R}(\frac{1}{T}-\frac{1}{{T}_{0}})\}$$

where *k*_0_ is the value of *k* at the reference temperature *T*_0_, *E* is the activation energy and *R* the gas constant. When data are collected over a wide enough temperature range, the contribution of the various terms in Eq. 2 change because the values of activation energies differ for processes that involve ligand binding, dissociation and conformational transitions. A global fit of the data resolves all individual rate constants and their associated activation energies, as shown by the results in Fig. 1C.

## Supplementary information

Structure of meizothrombin desF1

## Data Availability

Recombinant reagents and data presented in this study are available from the corresponding author upon reasonable request.
