# Supplementary material for: Residues W215, E217 and E192 control the allosteric E*-E equilibrium of thrombin
Source: Sci Rep. 2019 Aug 23;9:12304. doi: 10.1038/s41598-019-48839-1 (PMC6707225; doi:10.1038/s41598-019-48839-1)
Supplement: Supplementary file 1 — Structure of meizothrombin desF1 [file 41598_2019_48839_MOESM1_ESM.pdf]

SUPPLEMENTARY MATERIAL

Residues W215, E217 and E192 control the allosteric E*-E equilibrium of thrombin

Leslie A. Pelc, Sarah K. Koester, Zhiwei Chen, Noah E. Gistover and Enrico Di Cera

Edward A. Doisy Department of Biochemistry and Molecular Biology, Saint Louis University School of Medicine, St. Louis, MO 63104 USA

Figure S1 showing details of the active site region of the meizothrombin desF1 mutant S195A bound to PPACK, with the inhibitor acylating the catalytic H57. Density was contoured to 1 σ. Statistics for data collection and refinement are summarized in Table S1. Atomic coordinates and structure factors were deposited in the Protein Data Bank (accession code: 6PX5).

Table S1 summarizing the statistics for data collection and refinement of the structure of the meizothrombin desF1 mutant S195A bound to PPACK.

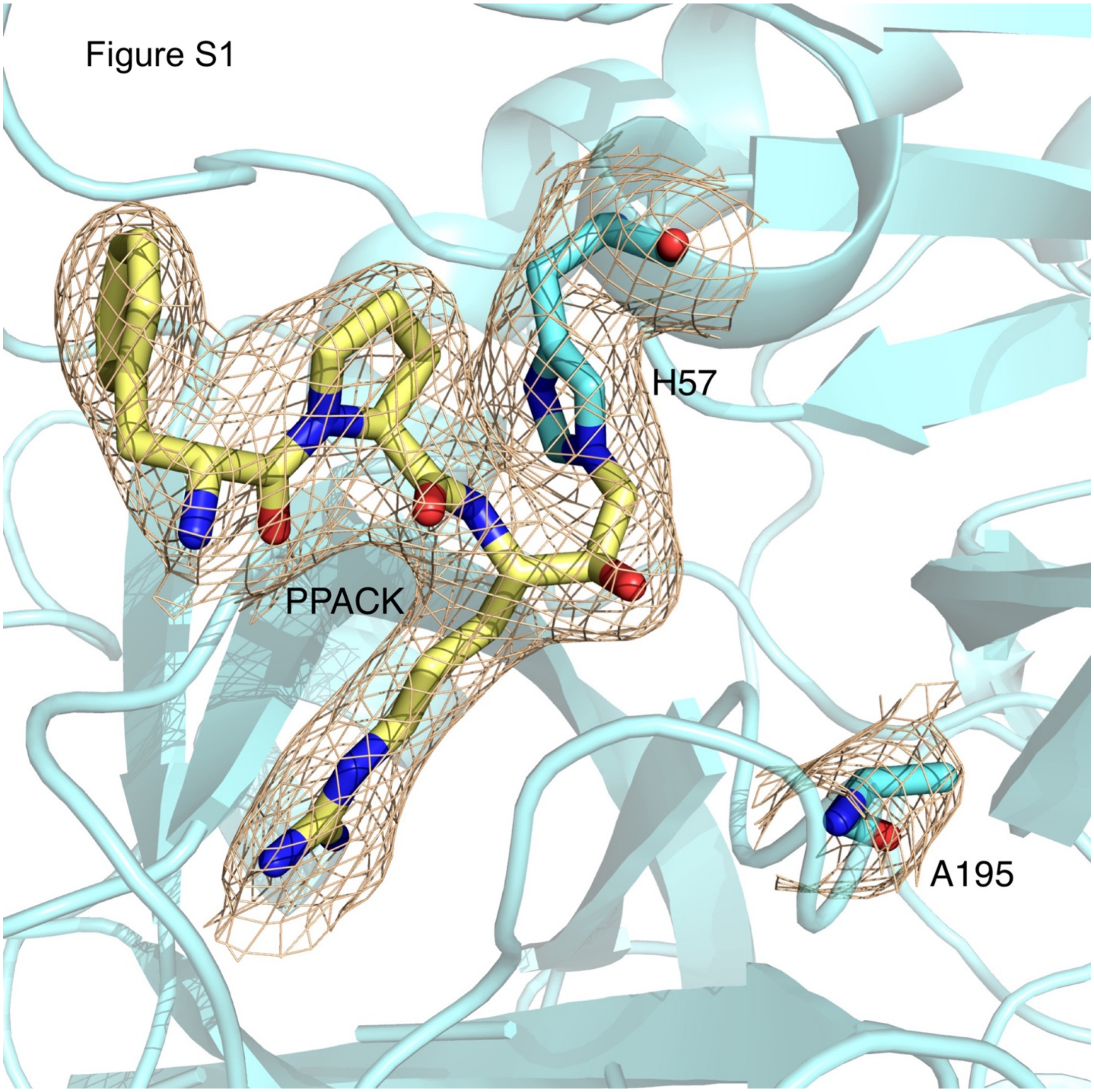
Figure S1
H57
PPACK
A195

Table S1: Crystallographic data for the meizothrombin desF1 mutant S195A bound to PPACK

PDB entry	6PX5
Buffer/salt	10 mM $ZnSO_4$, 100 mM MES, pH 6.5
PEG	550 MME (25%)
**Data collection:**	
Wavelength (Å)	1.54
Space group	$P3_221$
Unit cell dimensions (Å)	a=100.1, b=100.1, c=118.7
Molecules/asymmetric unit	1
Resolution range (Å)	40-2.4
Observations	196139
Unique observations	25684
Completeness (%)	94.1 (96.2)
R_{sym} (%)	10.0 (39.6)
$I/\sigma(I)$	15.7 (2.6)
**Refinement:**	
Resolution (Å)	40-2.4
R_{cryst}, R_{free}	0.22, 0.28
Reflections (working/test)	24293/1275
Protein atoms	3110
Na^+	1
PPACK	1
Zn^{++} ions	2
Solvent molecules	53
Rmsd bond lengths[a] (Å)	0.008
Rmsd angles[a] (°)	1.7
Rmsd ΔB ($Å^2$) (mm/ms/ss)[b]	6.17/5.30/6.04
<B> protein ($Å^2$)	81.7
<B> Na^+ ion ($Å^2$)	79.8
<B> PPACK ($Å^2$)	66.9
<B> Zn^{++} ions ($Å^2$)	74.7
<B> Solvent ($Å^2$)	67.8
**Ramachandran plot:**	
Most favored(%)	98.5
Generously allowed (%)	1.2
Disallowed (%)	0.3

[a]Root-mean-squared deviation (Rmsd) from ideal bond lengths and angles and Rmsd in B-factors of bonded atoms. [b]mm, main chain-main chain; ms, main chain-side chain; ss, side chain-side chain.
